# Dietary methionine deficiency affects oxidative status, mitochondrial integrity and mitophagy in the liver of rainbow trout (*Oncorhynchus mykiss*)

**DOI:** 10.1038/s41598-018-28559-8

**Published:** 2018-07-05

**Authors:** Sarah Séité, Arnaud Mourier, Nadine Camougrand, Bénédicte Salin, A. Cláudia Figueiredo-Silva, Stéphanie Fontagné-Dicharry, Stéphane Panserat, Iban Seiliez

**Affiliations:** 1 INRA, Univ Pau & Pays Adour, E2S UPPA, UMR 1419, Nutrition, Métabolisme, Aquaculture, Saint Pée sur Nivelle, F-64310 France; 2 Evonik Rexim, 80400 Ham, France; 3 Evonik Nutrition and Care GmbH, 63457 Hanau, Germany; 4 CNRS, IBGC, UMR5095, 1 rue Camille Saint-Saëns, F-33000 Bordeaux, France; 5 Université de Bordeaux, IBGC, UMR5095, 1 rue Camille Saint-Saëns, F-33000 Bordeaux, France; 6 Université de Bordeaux, Service Commun de Microscopie, 146 Rue Léo Saignat, F-33000 Bordeaux, France

## Abstract

The low levels of methionine in vegetable raw materials represent a limit to their use in aquafeed. Methionine is considered as an important factor in the control of oxidative status. However, restriction of dietary methionine has been shown to reduce generation of mitochondrial oxygen radicals and thus oxidative damage in liver. Here, we aim to evaluate the effect of dietary methionine deficiency in hepatic oxidative status in rainbow trout and identify the underlying mechanisms. Fish were fed for 6 weeks diets containing two different methionine concentrations: deficient (MD, Methionine Deficient diet) or adequate (CTL, control diet). At the end of the experiment, fish fed the MD diet showed a significantly lower body weight and feed efficiency compared to fish fed the CTL diet. Growth reduction of the MD group was associated to a general mitochondrial defect and a concomitant decrease of the oxidative status in the liver. The obtained results also revealed a sharp increase of mitochondrial degradation through mitophagy in these conditions and emphasized the involvement of the PINK1/PARKIN axis in this event. Collectively, these results provide a broader understanding of the mechanisms at play in the reduction of oxidant status upon dietary methionine deficiency.

## Introduction

Fisheries and aquaculture remain an essential resource for hundreds of millions of people worldwide. In 2014, the world fish supply reached 20 kg per capita, largely due to the strong development of aquaculture, which now provides 50% of the fish consumed^1^. Aquaculture is therefore an important player to consider in addressing one of the greatest challenges of our time: Feeding more than 9 billion people by 2050. However, the sustainability of this industry, which requires large amounts of wild fish for aquafeed, still remains a challenge^2^. Thus, the replacement of fishmeal and fish oil by proteins and oil of alternative origin is a major objective for sustainable aquaculture^3–6^. However, replacement of fish meal by plant proteins in aquafeed is often limited by the low level of methionine in those alternatives, in particular in plant protein sources. In this context, supplementation of agricultural crop sources with an appropriate level of synthetic methionine has been shown to optimize the nutritional value of those diets containing alternative proteins. A better understanding of the role of methionine in fish is therefore essential to develop diets that are in tune with fish growth and metabolism as well as environmental and economic constraints.

Besides its essential role as a building block for protein synthesis and growth of animals, methionine is proven a key factor in the control of oxidative status^7,8^. Indeed, methionine residues in proteins react readily with a variety of reactive oxygen species (ROS) to form methionine sulphoxide (MetO), and MetO reductases catalyze a thioredoxin-dependent reduction of MetO back to methionine *in vivo*. This cyclic interconversion of methionine residues of proteins between oxidized and reduced forms may therefore be considered as an efficient ROS-scavenging mechanism^9,10^. In addition, methionine feeds the transsulfuration pathway that leads synthesis of other sulfur amino acids, notably cysteine^11,12^. Cysteine is required for the synthesis of glutathione (GSH) and taurine, two components which have the capacity to affect cellular redox status and thus they are essential for host defense against oxidative stress^13–16^. Thus, methionine plays a primordial role in the defense against oxidative stress.

However, dietary methionine restriction has also been shown to decrease the rate of mitochondrial ROS production and thus oxidative damages. Indeed, rats maintained on an isocaloric 40% methionine restricted diet for 7 weeks have been shown to exhibit a decrease of heart mitochondrial ROS generation, particularly from complex Ι, as well as of damage to mitochondrial DNA, proteins, and lipids^17^. Similar results were obtained in liver, brain, and kidney mitochondria of animals fed 40% methionine restricted diet^18–21^. Likewise, decreases of mitochondrial ROS generation and oxidative stress were observed at 80% methionine restriction in both heart and liver^18,21^. However, the involved mechanisms remained far from being resolved. It has been hypothesized that reduction of mitochondrial ROS production observed in methionine restricted conditions is a direct result of a decrease of mitochondrial complex I levels^17–19,21,22^. Other possible explanatory mechanisms are decreased mitochondrial JO_2_, a mild electron transport chain uncoupling, or the substitution of mitochondrial membrane unsaturated fatty acids (20:4 n-6 and 22:6 n-3) by less unsaturated fatty acids (18:2 n-3, 18:1 n-9 and 18:0)^23^, suggesting that methionine restriction induces a more general mitochondrial dysfunction.

The present study was conducted to clarify the effect of dietary methionine deficiency in the control of hepatic oxidative status and identify the underlying mechanisms in rainbow trout (*Oncorhynchus mykiss*).

## Materials and Methods

The experiments were conducted in compliance with the legal frameworks of France and the EU. They respect the directive 2010/63/EU relating to the protection of animals used for scientific purposes as well as the decree No 2013-118, 1 February 2013 of the French legislation governing the ethical treatment of animals. The protocol was approved by the French National Consultative Ethics Committee under the reference number APAFIS8222-2017041016141425-v4.

### Feeding trial and rearing

Sexually immature rainbow trout *(Oncorhynchus mykiss)* having a mean initial weight of 60 g were reared in the INRA experimental facilities at Donzacq (Landes, France) at a constant water temperature (17 °C) under natural photoperiod during the months of July to August. The fish were distributed into 6 circular tanks (150 litres, 3 tanks per diet; 12 fishes/tank). Triplicate groups of trout were fed for 6 weeks with one of two iso-nitrogenous (40% crude protein) and isoenergetic (20 kJ/g dry matter, DM) extruded diets, manufactured at INRA experimental facilities of Donzacq (Table 1). Diets were formulated to meet nutrient requirements of rainbow trout (NRC 2011, AMINOSalmonid®) and thus be similar in their nutrient composition, except for methionine content that was intended to be adequate (0.93%) in the control diet (Control, CTL) or restricted by 56% (methionine deficient, MD) compared to the CTL diet. The cysteine (Cys) level was kept constant at 0.43 g Cys per 100 g diet. The diets were distributed *ad libitum* twice a day. Fish were counted and weighed at the beginning and end of the feeding trial to follow the growth and feed utilisation.

**Table 1:** Ingredient and analytical composition of the diets.

	CTL	MD
Ingredient (% dry weight)		
Fish protein concentrate^**a**^	5	5
Faba bean protein concentrate^**b**^	17.5	17.5
Soy protein concentrate^**c**^	17.5	17.5
White lupin meal^**d**^	12	12
Dehulled pea meal^**e**^	6	6
Fish oil^**f**^	15	15
Gelatinised starch^**g**^	10	10
CaHPO_4_.2H_2_O (18%P)	3	3
Min. premix. INR1^**h**^	2	2
Vit. premix. INRA^**i**^	2	2
Free amino acid	10*	10**
Analysed composition (%)		
Dry matter, DM	95.43	94.02
Carbohydrate (%DM)	14.58	14.62
Crude protein (% as fed)	42.15	41.92
Total lipid (%DM)	15.01	15.23
Gross energy (%DM)	22.78	22.83
*Amino acid (% as fed)*		
Cysteine	0.43	0.43
Histidine	1.26	1.24
Isoleucine	1.94	1.89
Leucine	3.61	3.54
Lysine	2.75	2.74
Methionine	0.93	0.41
Phenylalanine	2.15	2.12
Threonine	1.98	1.97
Valine	2.22	2.16
Alanine	2.52	2.59
Aspartic acid	3.77	3.77
Glutamic acid	5.40	5.41
Glycine	2.87	2.95
Proline	2.03	2.11
Serine	1.98	2.03

^a^CPSP-G (Sopropeche); ^b^Fabaqua 55 (Sotexpro); ^c^Estrilvo; CP 70 (Sopropêche); ^d^Farilup500 (Terrena); ^e^Aquatex (sotexpro); ^f^Southern hemisphere (Sopropêche); ^g^Roquette; ^h^Mineral premix (g or mg kg−1 diet): calcium carbonate (40% Ca), 4.3 g; magnesium oxide (60% Mg), 2.48 g; ferric citrate (21% Fe), 0.4 g; potassium iodide (76% I), 0.8 mg; zinc sulfate (36% Zn), 0.08 g; copper sulfate (25% Cu), 0.6 g; manganese sulfate (33% Mn), 0.06 g; dibasic calcium phosphate (23% Ca. 18%P), 10 g; cobalt sulfate, 0.4 mg; sodium selenite (46% Se), 0.6 mg; KCl, 1.8 g; NaCl, 0.8 g; ^i^Vitamin premix (IU or mg kg−1 diet): DL-a tocopherol acetate, 120 IU; sodium menadione bisulphate, 10 mg; retinyl acetate, 30.000 IU; DL-cholecalciferol, 6.000 IU; thiamine, 30 mg; riboflavin, 60 mg; pyridoxine, 30 mg; B12, 0.1 mg; nicotinic acid, 350 mg; folic acid, 1 g; inositol, 2 g; biotin, 5 mg; calcium pantothenate, 0.1 g; choline chloride, 4 g; *Free amino acid (% of the diet): Arginine, 0.07%; Cysteine, 0%; Histidine, 0.42%, Isoleucine, 0.30%; Leucine, 0.77%; Lysine, 1.36%; DL-methionine0.55%; Phenylalanine, 0.5%; Threonine, 0.81%; Tryptophan, 0.07%; Tyrosine, 0.45%; Valine, 0.54%; Alanine, 0.97%, Aspartic acid, 0.51%; Glutamic acid, 0.45%; Glycine, 1.20%; Proline, 0.52%, Serine, 0.5% (Evonik); **Free amino acid (% of the diet): Arginine, 0.07%; Cysteine, 0%; Histidine, 0.42%, Isoleucine, 0.30%; Leucine, 0.77%; Lysine, 1.36%; DL-methionine0.0%; Phenylalanine, 0.5%; Threonine, 0.81%; Tryptophan, 0.07%; Tyrosine, 0.45%; Valine, 0.54%; Alanine, 1.10%, Aspartic acid, 0.58%; Glutamic acid, 0.51%; Glycine, 1.35%; Proline, 0.58%, Serine, 0.57%.

Individual body mass, daily growth index, daily feed intake and feed efficiency were calculated as described before^24^.

At the end of the 6 weeks feeding trial, blood and liver were collected from 3 fish per tank, anesthetised with benzocaine (30 mg/L) and euthanized by a sharp blow to the head. Blood samples were collected 16 h after the last meal from the caudal vein into heparinized syringes and centrifuged (3000 g, 5 min); the recovered plasma was immediately frozen and kept at −20 °C. Livers were collected at 2 h, 4 h and 16 h after the last meal, dissected, weighted and immediately frozen in liquid nitrogen and kept at −80 °C.

### Chemical composition of the diets

DM, crude fat and gross energy content of the diets were determined following the procedures previously outlined^24^. Crude protein content was measured as N × 6.25 by the Kjeldahl method after acid digestion (ISO 937:1978) and using Leco FP-2000 (Leco Corp., St. Joseph, MI) analyser. Starch content was measured by an enzymatic method (InVivo Labs). Dietary amino acid concentrations were performed by wet chemistry at Evonik-Degussa Laboratory (Hanau, Germany) by ion-exchange chromatography with postcolumn derivatization with ninhydrin. Amino acids were oxidized with performic acid, which was neutralized with sodium metabisulfite^25^; Commission Directive 1998). Amino acids were liberated from the protein by hydrolysis with 6 N HCl for 24 h at 110 °C and quantified with the internal standard method by measuring the absorption of reaction products with ninhydrin at 570 nm.

### Level of methionine in plasma

Free methionine concentrations in blood plasma were determined by ion exchange chromatography using a Biochrom 20 amino acid analyser Lithium column and lithium buffers^26,27^.

### Western blot analyses

Livers sampled 2 and 16 h after the last meal were homogenized and analysed according to the previously detailed protocol^24^ and using the following antibodies: anti-phospho- ribosomal protein S6 kinase 1 (P- RPS6K1, Thr389, #9205, Cell Signaling Technology); anti- RPS6K1 (#9202, Cell Signaling Technology); phospho-EIF4EBP1, eukaryotic translation initiation factor 4E binding protein 1 (P- EIF4EBP1, Thr37/Thr46, #9459, Cell Signaling Technology); anti- EIF4EBP1 (#9452, Cell Signaling Technology); anti-phospho eIF2α (Ser51, #9721, Cell Signaling Technology); anti-carboxyl terminal eIF2α (#9722, Cell Signaling Technology); anti-phospho AMP-activated protein kinase (P-AMPK, Thr172, #2532, Cell Signaling Technology); anti-AMPK (#2532, Cell Signaling Technology); anti-β-tubulin (TUBB)(#2146, Cell Signaling Technology); anti-LC3B (#2775, Cell Signaling Technology). All these antibodies have already been validated in rainbow trout^24,28^.

To assess the levels of mitochondria-related proteins (TIMM23, mitofusin2, PARKIN, phospho-Ubiquitin (Ser65) and total Ubiquitin), tissues were homogenized by ultrasonic disruption in 9 vol of ice-cold buffer containing 50 mM Tris (pH 7.4), 5 mM EDTA, 2 mM 1,4-dithiothreitol, and a protease inhibitor cocktail (Sigma, St. Louis, MO; P-2714). Then, the homogenate was centrifuged, and the supernatant was used immediately for western blot analysis as described above and using the appropriate antibodies: anti-TIMM23 (#611222, BD Transduction Laboratories^TM^), anti-mitofusin2 (Mfn2) (#ab56889, abcam), anti-PARKIN (#ab15954, abcam), anti-phospho-Ubiquitin (Ser65) (Ser65, #ABS1513-I, EMD Millipore) and anti-total Ubiquitin (#MAB1510, EMD Millipore). Before the use of each antibody, the amino acid sequence of the targeted protein was monitored in the SIGENAE database (http://www.sigenae.org) to check for the conservation of the antigen sequence with the corresponding sequence from mammals, ensuring a good specificity of the mammalian antibody used in the analysis of the samples.

### Enzyme activity

Livers collected 16 h after the meal were homogenised with a potter at 4 °C, in a buffer containing 0.1 M of triethanolamine, pH 7.3. The homogenate was then used for enzymatic assays. Protein concentration was measured using the Lowry’s method.

Proteins (50–1500 µg) were diluted in triethanolamine (0.1 M triethanolamine, pH 7.3) followed by spectrophotometric analysis of enzymatic activities at 37 °C using a spectrophotometer. Citrate synthase activity was measured at 412 nm (ε = 13,600 M^−1^cm^−1^) after the addition of 0.1 mM acetyl-CoA, 0.1 mM oxaloacetate, and 0.1 mM 5, 5-dithiobis-2-nitrobenzoic acid (DTNB). Succinate dehydrogenase (SDH or Complex II) activity was measured at 600 nm (ε = 21 mM^−1^cm^−1^) after the addition of 0.1 mM phenazine methosulphate (PMS), 40 mM succinate, 0.1 mM dichlorophenolindophenol (DCPIP) and 40 mM malonate. The specific Complex II activity was defined as the flux difference before and after the addition of 40 mM malonate (Complex II inhibitor). NADH ubiquinone oxydo-reductase (Complex I) activity was measured at 340 nm (ε = 6220 M^−1^.cm^−1^) after the addition of 0.5 mM decylubiquinone, 4.05 µg.ml^−1^ bovine serum albumin (BSA), 0.5 mM antimycin, 0.5 mM NADH and 12.5 µM rotenone. The specific Complex I activity was defined as the flux difference before and after the addition of rotenone (inhibitor of Complex I). Cytochrome *c* oxydase (Complex IV) was determined at 550 nm (ε = 18,500 M^−1^cm^−1^) after the addition of 1 µM antimycin, and 100 µM cytochrome c. The specific Complex IV activity was defined as the flux difference before and after the addition of 1 mM KCN (inhibitor of Complex IV). Aconitase activity was measured using the Aconitase Enzyme Activity Assay kit (ab109712; Abcam; Cambridge, MA) according to manufacturer’s instructions.

Catalase activity was assessed using an Oroboros oxygraph. Catalase activity of homogenized tissues was followed by recording the oxygen production in the presence of 0.01% H_2_O_2_ and 1 µM antimycin to prevent the interfering mitochondrial oxygen consumption. All enzyme activities are expressed as percentage of control condition, apart from Catalase which was expressed as O_2_ flux pmol/s*ml*mg.

### Quantitative RT-PCR analyses

Quantitative RT-PCR analyses were performed on liver of fish sampled at 2 and 16 h after the last meal. The protocol conditions for sample preparation and quantitative RT-PCR have been previously published^28,29^. The primers used for real-time RT-PCR assays are listed in Table 2. Primers of *peroxisome proliferator-activated receptor-γ coactivator-1α (pgc1α)*, were newly designed using Primer3 software. For the expression analysis, relative quantification of target gene expression was done using the ΔCT method described by^30^. The relative gene expression value *of eukaryotic translation elongation factor 1 α 1 (eef1a1)* was used for the normalization of the measured expression values of the target mRNA, and it was found to not change significantly over sampling time or among dietary treatments (data not shown).

**Table 2:** Sequences of the primer pairs used in the quantitative real-time RT-PCR assays.

Genes	Forward primer	Reverse primer
**GCN2-eif2α target genes**		
*Asns*	CTGCACACGGTCTGGAGCTG	GGATCTCGTCTGGGATCAGGTT
*Ddit3*	CTGCACACGGTCTGGAGCTG	GGATCTCGTCTGGGATCAGGT
**Antioxidant defence**		
*sod1*	TGGTCCTGTGAAGCTGATTG	TTGTCAGCTCCTGCAGTCAC
*sod2*	TCCCTGACCTGACCTACGAC	GGCCTCCTCCATTAAACCTC
gstπ	TCGCTGACTGGACGAAAGGA	CGAAGGTCCTCAACGCCATC
*gsr*	CTAAGCGCAGCGTCATAGTG	ACACCCCTGTCTGACGACAT
**Mitochondrial synthesis**		
*Pgc1α*	CCCCAGAGTCTCCAAATGAC	GGTGTCAGACCTGGGGTTC
**Reference gene**		
*eef1a1*	TCCTCTTGGTCGTTTCGCT	ACCCGAGGGACATCCTGTG

### Determination of oxidative status

Protein carbonyls and glutathione levels were measured in fish liver sampled 16 h after the last meal (N = 6/diet). Samples were prepared and analyzed for protein carbonyls according to the manufacturer’s protocol (Millipore; Oxyblot Protein Oxidation Detection Kit S 7150). Protein quantification was carried out with the Odyssey software expressing the ratio of DNPH-derivatized proteins/β-tubulin (TUBB) of the liver.

Oxidized glutathione (GSSG) and reduced glutathione (GSH) were measured in fish liver homogenates using Cayman glutathione assay kit (Bertin Pharma) according to the manufacturer’s instructions.

### Determination of mitochondrial DNA

DNA isolation was performed on fish liver sampled 16 h after the last meal (N = 6/diet) following the procedures previously outlined in Liu *et al*.^31^. mtDNA copy number was determined using quantitative PCR Gene abundance was detected as described above in quantitative RT-PCR section. mtDNA copy number (mitochondrially encoded tRNA leucine 1^UUA/G^: Forward primer: AAAACAGACAAGGGGGCACA, Reverse primer: AGGGTGAGGAAAGCAACTGC) was normalized to Beta-2-macroglobulin genomic DNA in the sample (Forward primer: TCATCGCTGCAGGTGTGTAT, Reverse primer: TAAATATCGCCCAGCCAAAC). Primers of the MT-TL1 and beta 2 M genes were newly designed using Primer3 software and the obtained amplicons were purified and sequenced (Beckman-Coulter Genomics, Takeley, UK).

### Electron microscopy

For electron microscopy, fresh liver blocks, sampled 4 h after the last meal (1 mm^3^) (N = 3/diet) were fixed with 2.5% glutaraldehyde in 0.1 M phosphate buffer (pH 7.4), and post-fixed in 1% osmium tetroxide in phosphate buffer. Conditions of both the treatments of ultrathin sections and observations were already described in Seiliez *et al*.^28^.

### Statistical analyses

Data are expressed as means ± SEM. Normality was assessed using the Shaprio–test, while the equality of variances was determined using Levene’s test. When the normality and/or equal variances of data were respected, Student’s t-test was used for comparison of data between fish fed CTL diet and fish fed MD diet. In contrast, when data did not meet the assumptions of normality and/or equal variances, Wilcoxon test was used to compare data from the two groups. Statistical analyses of the mRNA levels of the genes *Asns* and *ddit3* were carried out using two-way ANOVA. Statistical analyses were performed using R software. For all statistical analyses, the level of significance was set at P < 0.05.

## Results

### Growth performance

At the end of the feeding trial, the final body weight and feed efficiency (but not the feed intake) were significantly lower in fish fed the MD diet than in those fed the CTL diet (Table 3). Hepatosomatic index (HSI), *i.e*. the liver to body weight ratio, used as a marker of methionine deficiency stress, significantly increased in methionine deficiency group. Moreover, as expected, plasma levels of methionine were significantly lower in trout fed the MD compared to the CTL diet (2.31 vs 0.06).

**Table 3:** Effect of methionine restriction in rainbow trout on weight (g), feed intake (% body weight/d), feed efficiency, hepatosomatic index (HIS) and 16 h postprandial plasma methionine level.

	CTL		MD	
	Mean	SEM	Mean	SEM
Initial body weight(g)	63.97	1.53	61.14	1.26
Final body weight (g)	137.33^a^	3.77	99.97^b^	3.49
Daily growth index (%/d)	3.63^a^	0.29	2.13^b^	0.11
Feed intake (%body weight/d)	1.86	0.02	1.93	0.09
Feed efficiency	1.19^a^	0.07	0.75^b^	0.01
HSI	0.94^a^	0.02	1.75^b^	0.01
Plasma methionine level (mg/ml)	2.31^a^	0.65	0.06^b^	0.01

CTL, control diet; MD, methionine deficient diet. Daily growth index = 100 × (mean final body mass^1/3^ – mean initial body mass^1/3^)/day. Feed intake = 100 × the total amount of ingested feed (kg) divided by the mean biomass over the experimental period ((initial biomass + final biomass)/2), expressed as kg wet mass) and the number of days. Feed efficiency = Gain in total biomass [(final biomass – initial biomass) (kg wet mass)] divided by the amount of ingested dry matter (kg DM). HSI = (liver weight/total body weight) × 100. ^a,b^Mean values with unlike superscript letters were significantly different among the two dietary groups (P < 0.05; t-test).

### Effect of methionine deficiency on key factors of the nutrient sensing pathways mTOR and GCN2/eIF2α

**Figure 1:**
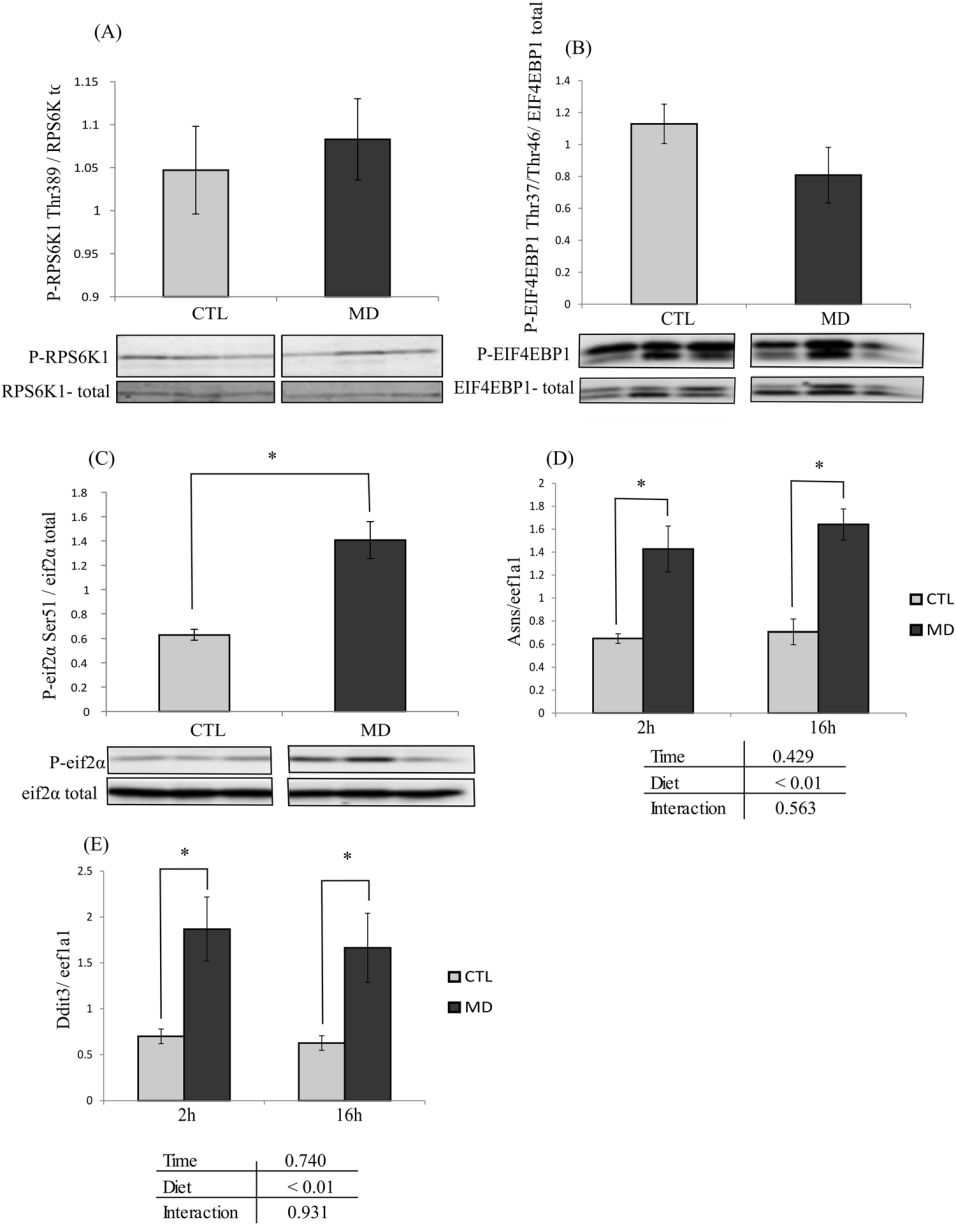
Effect of methionine deficiency on key factors of the mTOR and GCN2/eIF2α signaling pathways Phosphorylation of (**A**), ribosomal protein S6 kinase 1 (RPS6K1) (**B**) eukaryotic translation initiation factor 4E binding protein 1 (EIF4EBP1) and (**C**) eukaryotic translation initiation factor 2α (eIF2α) proteins in liver of trout fed the control diet (CTL) or the methionine deficient diet (MD) and sampled 2 h after the last meal. Western blot analysis was carried out on six individual samples per treatment, and a representative blot is shown (Source data for each blot are available in Supplementary Fig. 1). Graphs show the ratio of the amount of the phosphorylated protein: the total amount of the targeted protein. The mRNA levels of (**D**) *asparagine synthetase* (*asns*) and (**E**) *DNA-damage inducible transcript 3* (*ddit3*) were measured using quantitative real-time RT-qPCR assays in liver 2 and 16 h after the last meal. Expression values are normalized with the *eukaryotic translation elongation factor 1 α 1 (eef1a1)* mRNA. Values are means (n = 6), with standard error of the mean represented by vertical bars. *was used to indicate significant difference between treatment among the two dietary group (P < 0.05; one-way ANOVA followed by the student-Newman-Keuls multiple-comparison test).

As shown in Fig. 1 (panels A and B), the phosphorylation of the two mTOR effectors RPS6K1 and EIF4EBP1 were not different among the two dietary groups. In contrast, the phosphorylation of eIF2α as well as the mRNA levels of its two target genes *asns* and *ddit3* (analysed 2 h and 16 h after the last meal), were significantly increased in fish fed the MD diet compared to the control group (Fig. 1C–E). These results indicated that the methionine deficiency was efficiently sensed at the cellular level in fish fed the MD diet.

### Methionine deficiency decreases the hepatic oxidative status

**Figure 2:**
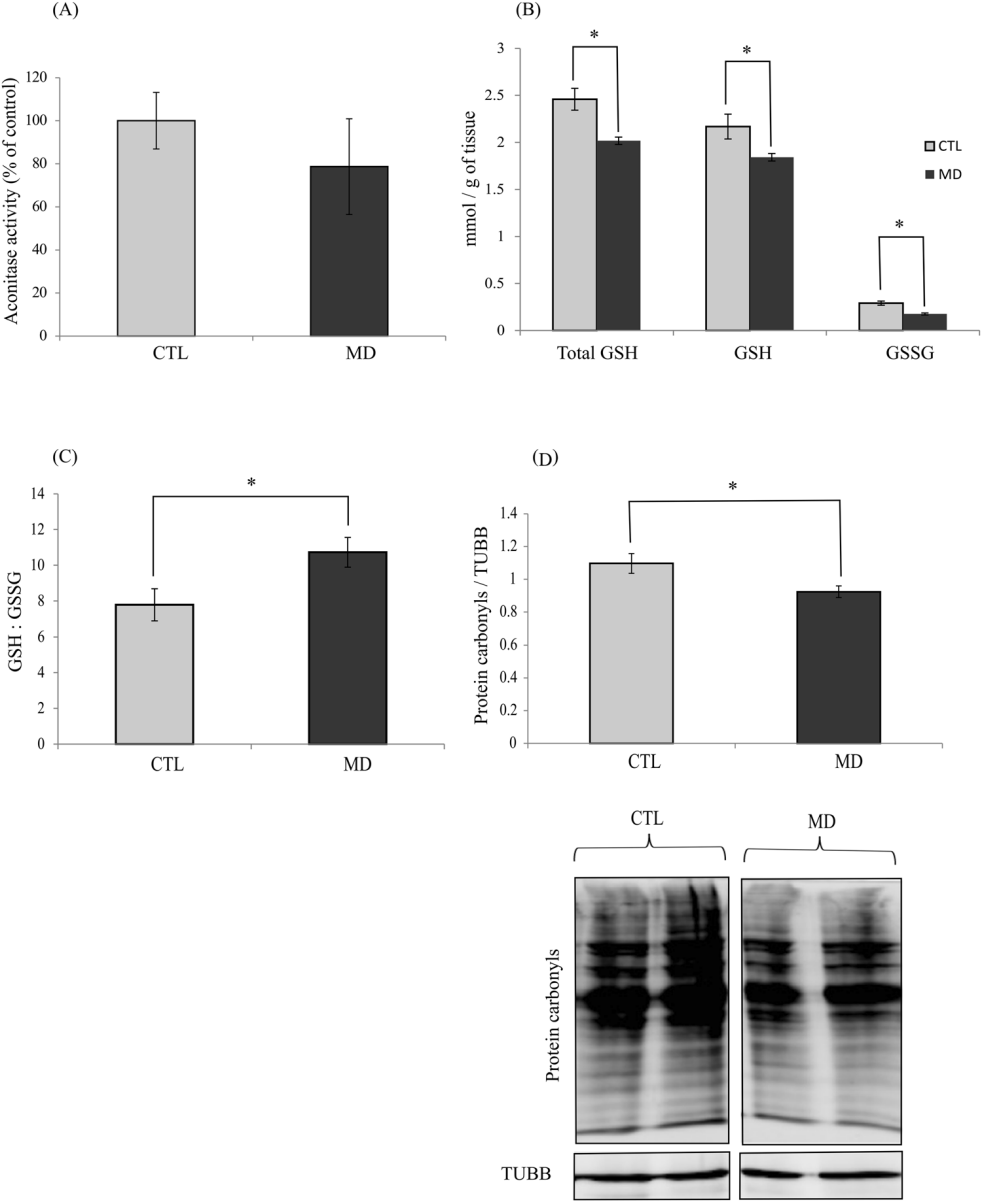
Methionine deficiency decreases the oxidative status. (**A**) Aconitase activity, (**B**) quantity of total glutathione (total GSH), reduced glutathione (GSH) and oxidized glutathione (GSSG), (**C**) ratio GSH/GSSG, (**D**) levels of protein carbonyls in liver of trout fed control diet (CTL) or deficient diet (MD) and sampled 16 h after the last meal. Levels of protein carbonyls were measured by Oxyblot. DNP-derivatized liver tissue lysates were analysed by Western blot for the presence of oxidized protein. Western blot analysis was carried out on six individual samples per treatment, and a representative blot is shown (Source data are available in Supplementary Fig. 2). Graphs show the ratio of the amount of the oxidized protein: β-tubulin (TUBB) used as a loading control. Values are means (n = 6), with standard error of the mean represented by vertical bars. *was used to indicate significant difference between treatment among the two dietary group (P < 0.05; t-test).

Considering that methionine is involved in the antioxidant defence system, we measured several oxidative stress markers. A drop in Aconitase activity has been described in abnormal situations, such as increased oxidative stress. Here, the activity of Aconitase was not significantly impacted by the methionine level in the diet (Fig. 2A). However, interestingly, hepatic glutathione levels of both reduced (GSH) and oxidized (GSSG) forms were significantly lower in fish fed the MD compared to the CTL diet (Fig. 2B). The content of GSH and GSSG were decreased respectively by 1.2 and 1.7 folds, which resulted in a higher GSH/GSSG ratio in MD than CTL groups (Fig. 2C), supporting a decrease of the oxidative status in fish fed the MD diet. In agreement with these results, the level of protein carbonyls, which is a marker of protein oxidation, was significantly lower in the liver of fish fed the MD diet compared to the control fish (Fig. 2D).

### Methionine deficiency has no impact on antioxidant defence

**Figure 3:**
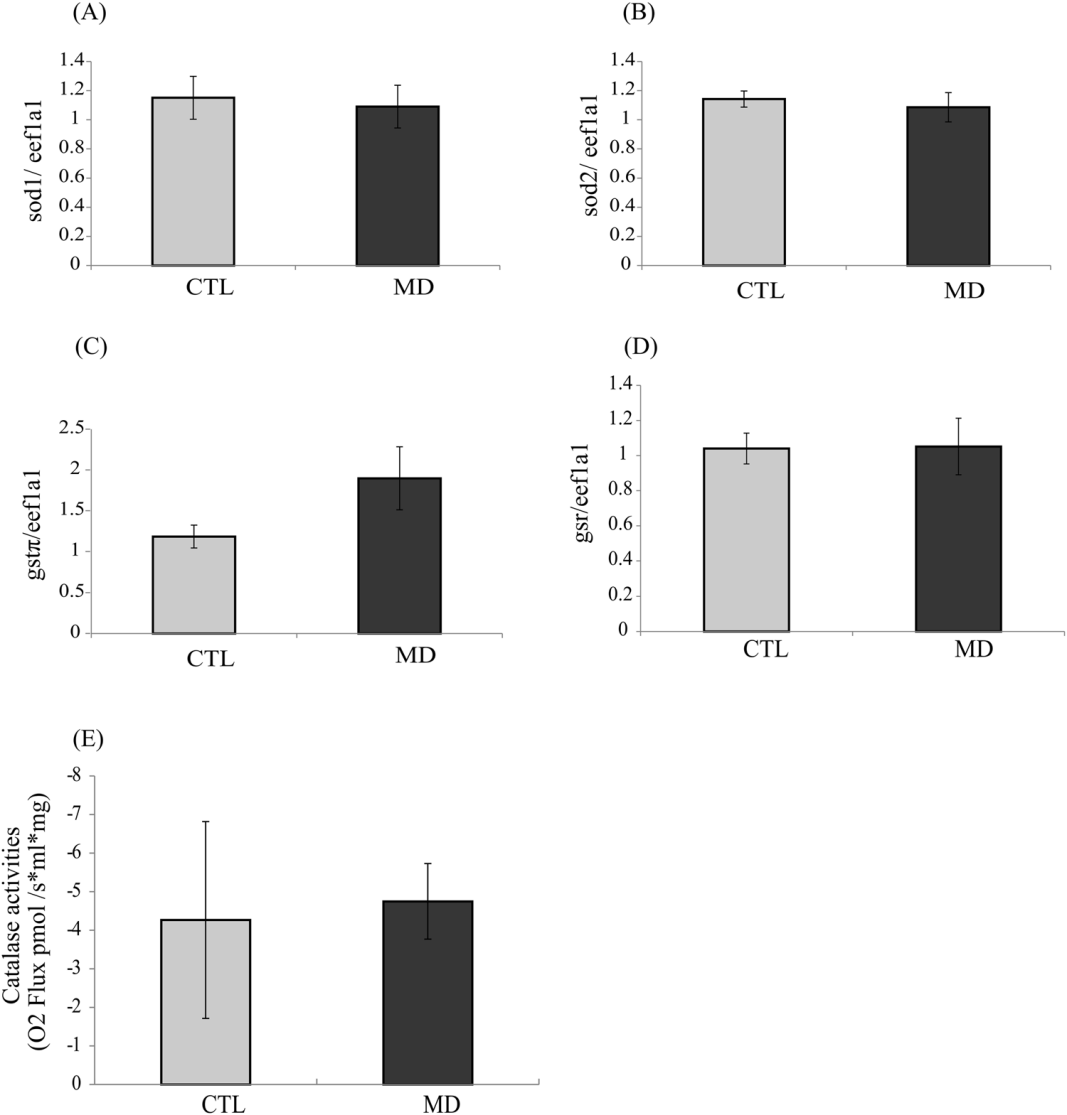
Methionine deficiency has no impact on antioxidant defence The mRNA levels of (**A**) *superoxide dismutase 1* (*sod1*), (**B**) *superoxide dismutase 2* (*sod2*), (**C**) *glutathione-S-transferase π* (*gstπ*) and (**D**) *glutathione-disulfide reductase (gsr*) were measured using quantitative real-time RT-qPCR assays in liver of trout fed control (CTL) or methionine deficient diet (MD) and sampled 16 h after the last meal. Expression values were normalized with the *eukaryotic translation elongation factor 1 α* 1 *(eef1a1*) mRNA. Catalase activity (**E**) was measured by oxygraphe 16 h after the last meal. Values are means (n = 6), with standard error of the mean represented by vertical bars. *was used to indicate significant difference between treatment among the two dietary group (P < 0.05; t-test).

In order to investigate if the observed decrease of oxidative status in fish fed the MD diet was due to an increase of antioxidant defence, we assessed the mRNA levels of four genes involved in antioxidant defence: mitochondrial *superoxide dismutase 2* (*sod2*); cytosolic *superoxide dismutase 1* (*sod1*), *glutathione-disulfide reductase* (*gsr)* and *glutathione-S-transferase π* (*gstπ*). As shown in Fig. 3, the levels of *sod1*, *sod2*, *gstπ* and *gsr* transcripts were not different among the two dietary groups. Moreover, we measured the activity of the anti-oxidant enzyme Catalase and observed no significant differences among the two dietary groups. All these results suggested that methionine deficiency do not impact the antioxidant defence system.

### Methionine deficiency has an impact on oxidative phosphorylation and induce energetic stress

**Figure 4:**
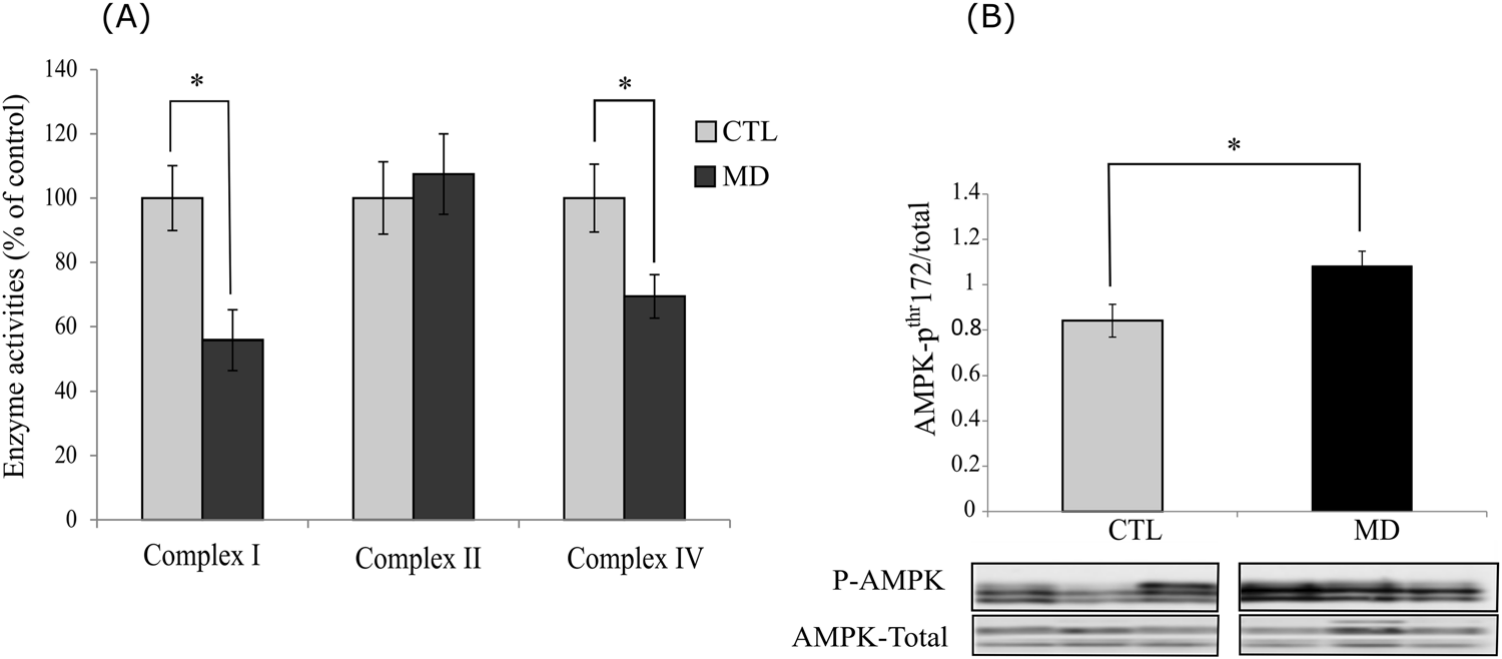
Methionine deficiency affects oxidative phosphorylation and induces energetic stress (**A**). Activities of Complex I, Complex II and Complex IV of OXPHOS system in the liver of trout fed the control diet (CTL) or the methionine deficient diet (MD) and sampled 16 h after the last meal. Analysis was carried out on six individual samples per treatment. (**B**) Phosphorylation of AMPK in the liver of trout fed the control diet (CTL) or the methionine deficient diet (MD) and sampled 16 h after the last meal. Western blot analysis was carried out on six individual samples per treatment, and a representative blot is shown (Source data are available in Supplementary Fig. 3). Graph show the ratio of the amount of the phosphorylated protein: the total amount of the targeted protein. Values are means (n = 6), with standard error of the mean represented by vertical bars. *was used to indicate significant difference between treatment among the two dietary group (P < 0.05; t-test).

Mitochondria are commonly described as an important source of ROS generation through electron leakage from their respiratory chain. Therefore, we assessed activities of different complexes of the respiratory chain (I, II and IV). As shown in Fig. 4A activity of Complex II was not impacted by methionine deficiency. In contrast, activities of both the Complex I and IV decreased in fish fed the MD diet compared to those of fish fed the CTL diet. Moreover, we observed an increase of the phosphorylation of the energy sensing factor AMPK in the liver of fish fed the MD diet supporting a fall in the energy status in this condition (Fig. 4B).

### Methionine deficiency and mitochondrial mass

**Figure 5:**
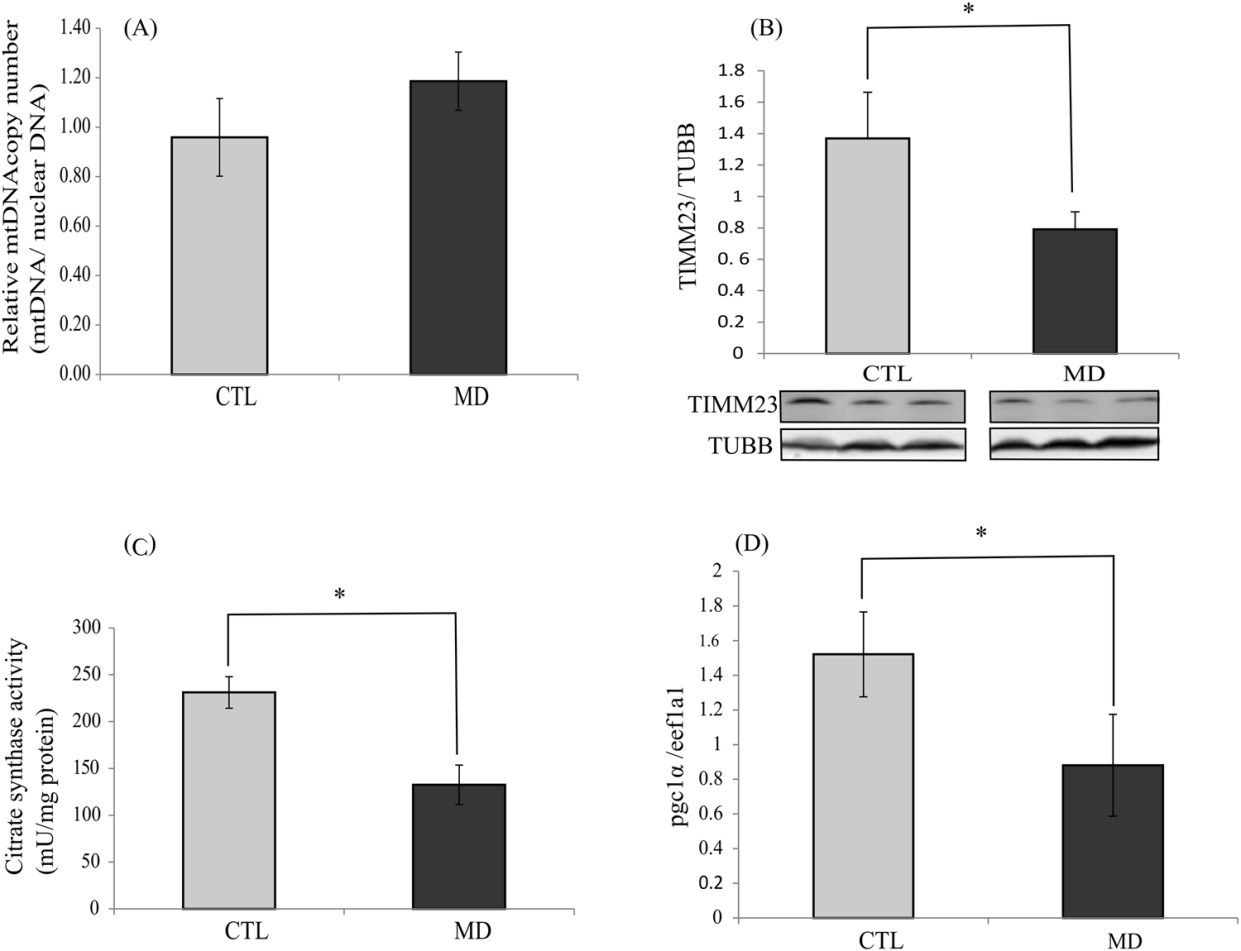
Methionine deficiency and mitochondrial mass. (**A**) Relative mtDNA copy number in the liver of trout fed the control diet (CTL) or the methionine deficient diet (MD) and sampled 16 h after the last meal. (**B**) TIMM23 levels in the liver of trout fed the control diet (CTL) or the methionine deficient diet (MD) and sampled 16 h after the last meal. Western blot analysis was carried out on six individual samples per treatment, and a representative blot is shown (Source data are available in Supplementary Fig. 4). Graphs show the ratio of the amount of TIMM23: β-tubulin (TUBB) used as a loading control. (**C**) Citrate synthase activity in the liver of trout fed the control diet (CTL) or the methionine deficient diet (MD) and sampled 16 h after the last meal. Analysis was carried out on six individual samples per treatment. (**D**) mRNA level of *pgc1α* in the liver of trout fed the control diet (CTL) or the methionine deficient diet (MD) and sampled 16 h after the last meal. Expression values are normalized with the *eukaryotic translation elongation factor 1 α 1 (eef1a1)* mRNA. Values are means (n = 6), with standard error of the mean represented by vertical bars. *was used to indicate significant difference between treatment among the two dietary group (P < 0.05; t-test or Wilcoxon test).

In order to determine whether the observed fall of the activity of the OXPHOS complexes I and IV as well as of the phosphorylation of AMPK were due to a decrease in mitochondrial mass, we measured four mitochondrial markers: the relative mitochondrial DNA copy number, the levels of TIMM23 (a protein located in mitochondrial inner membrane), the activity of citrate synthase (involved in the citric acid cycle and localized in the mitochondrial matrix) and the mRNA levels of the *peroxisome proliferator-activated receptor-γ coactivator-1α* (*pgc1α*) which is considered to be a major regulator of mitochondrial biogenesis. The obtained results show that the relative mitochondrial DNA copy number was not different among the two dietary groups (Fig. 5A). In contrast, fish fed the MD diet exhibited a significant decrease of TIMM23, citrate synthase activity and *pgc1α* mRNA levels (Fig. 5B–D). Together, these data support a disturbance in the mitochondrial function rather than a fall of the mitochondrial mass in liver of fish fed the MD diet.

### Methionine deficiency lead to induction of mitophagy

**Figure 6:**
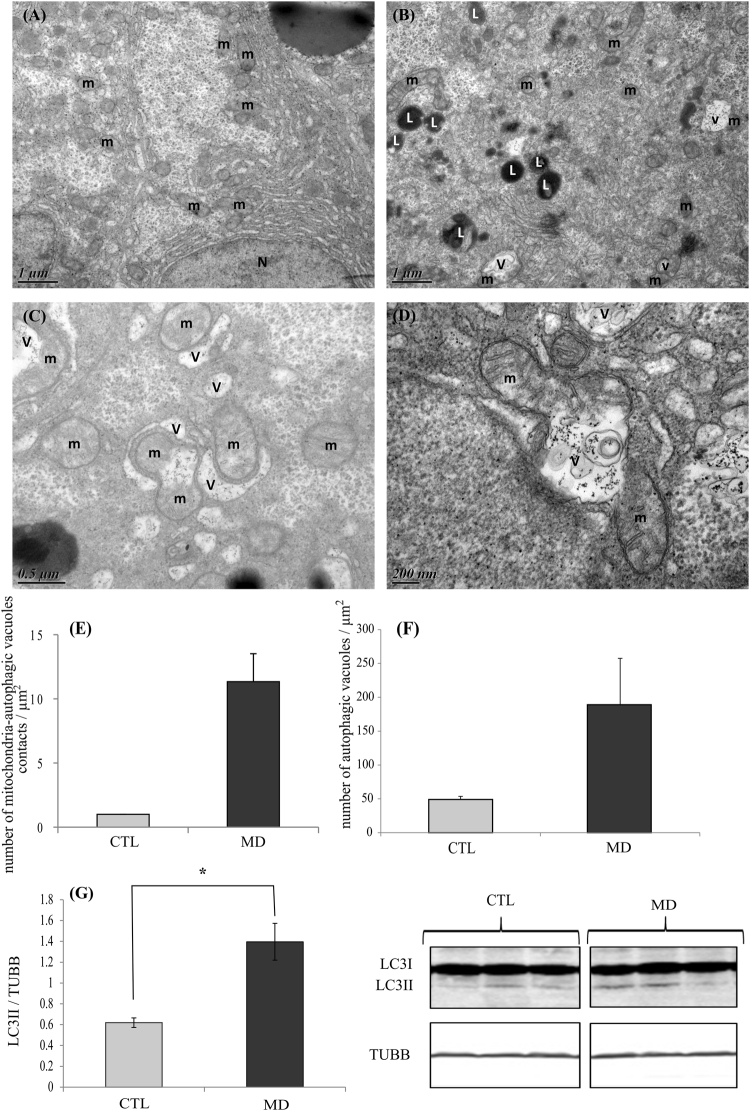
Methionine deficiency lead to induction of mitophagy. Electron microscopy (EM) analysis of liver section of trout fed the control diet (CTL) or the methionine deficient diet (MD). Picture (**A**) represents liver sections of trout fed MD diet and picture (**B**–**D**) liver section of trout fed deficient diet. N, nucleus; m, mitochondria; v, autophagic vacuoles and L, lysosome. Mitophagy is demonstrated by the presence of mitochondria engulfed and/or being engulfed inside autophagosome-related vacuoles. The graphe (**E**) represents the quantity of mitochondria-autophagic vacuoles in contact and (**F**) the quantity of autophagic vacuoles per µm2 in the EM images (n = 3 samples with 8 to 10 micrographs per sample). (**G**) LC3II protein in liver of trout fed CTL diet or MD diet and sampled 4 h after the last meal. Western blot analysis was carried out on six individual samples per treatment, and a representative blot is shown (Source data are available in Supplementary Fig. 5). Graphs show the ratio of the amount of LC3II: β-tubulin (TUBB) used as a loading control. Values are means (n = 6), with standard error of the mean represented by vertical bars. *was used to indicate significant difference between treatment among the two dietary group (P < 0.05; t-test or Wilcoxon test).

Transmission electron microscopy on liver samples revealed a sharp increase of mitochondria engulfed and/or being engulfed inside autophagosome-related vacuoles (a process called mitophagy) in fish fed the MD diet (Fig. 6B–E). Moreover, we also found that the total number of autophagosome-related vacuoles (with and without mitochondria) was most abundant in the liver of fish fed the MD diet (Fig. 6F). Accordingly, the autophagosomal marker LC3-II exhibited significantly higher levels in trout fed the MD diet (Fig. 6G). Overall, these results suggested that methionine deficient diet lead to increase mitochondrial degradation via mitophagy.

### Effect of methionine deficiency on the PINK1/PARKIN axis

**Figure 7:**
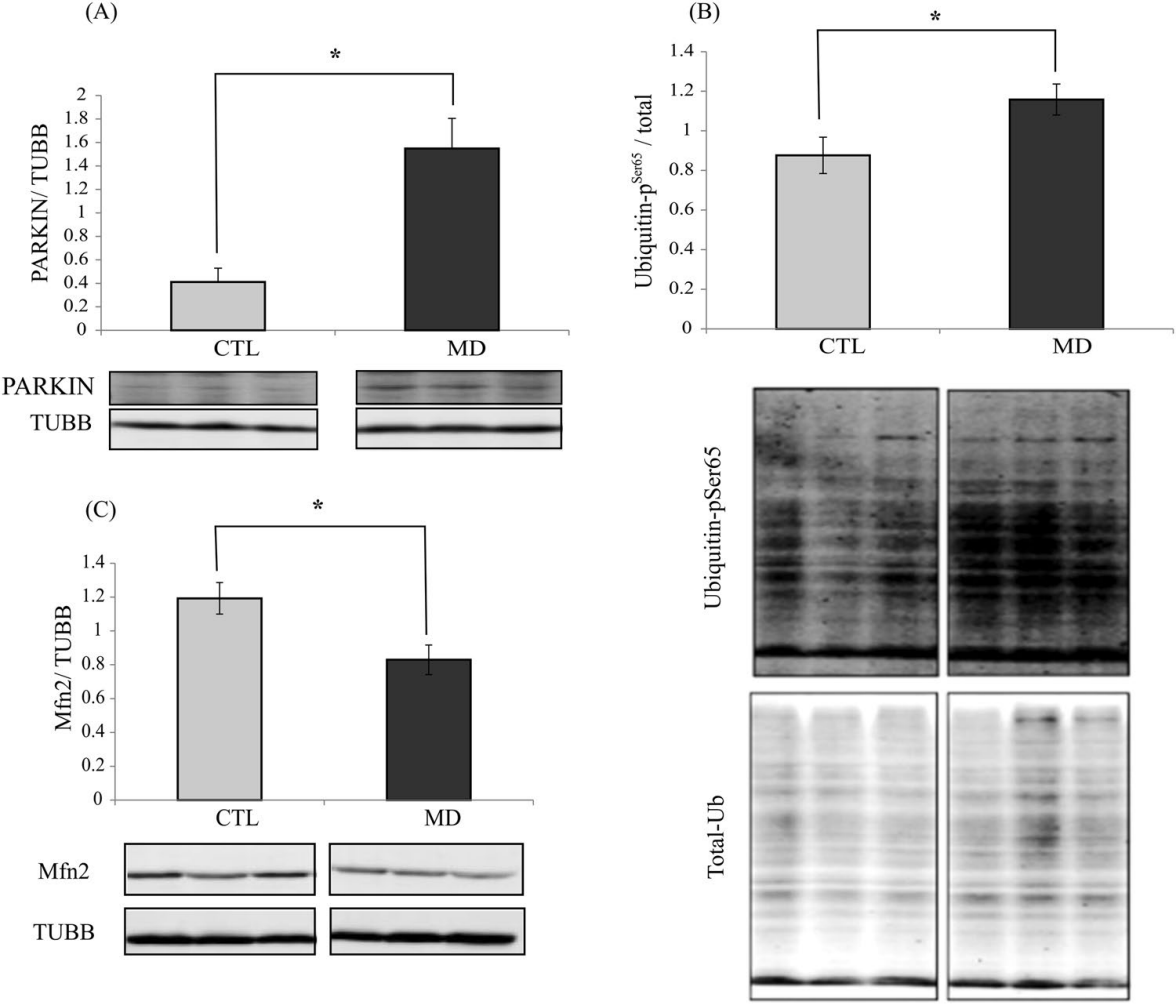
Methionine deficiency leads to induction of the PINK11/PARKIN axis. Western blot analysis of (**A**) PARKIN, (**B**) Ubiquitin-p^Ser65^ and (**C**) Mfn2 in the liver of trout fed the control diet (CTL) or methionine-deficient diet (MD) and sampled 16 h after feeding the last meal. Western blot analysis was carried out on six individual samples per treatment, and a representative blot is shown (Source data for each blot are available in Supplementary Figs 6 and 7). Graphs show the ratio of the targeted protein: β-tubulin (TUBB) or the total amount of the targeted protein used as a loading control. Values are means (n = 6), with standard error of the mean represented by vertical bars. *was used to indicate significant difference between treatment among the two dietary group (P < 0.05; t-test).

PINK1 (PTEN induced putative kinase 1)/PARKIN (parkin RBR E3 ubiquitin protein ligase) axis is considered as a major pathway of mitophagy regulation^32^. Induction of this pathway has been shown to correlate with an increase of ubiquitin phosphorylation at Ser65 (p-S65-Ub) and a decrease of the level of the PARKIN-target protein Mfn2^33^. As shown in Fig. 7A–C, there were large and significant increases of both PARKIN and p-S65-Ub levels, and a concomitant decrease of Mfn2 in the liver of fish fed the MD diet at 16 h post-feeding. Thus, these results identified the PINK1/PARKIN axis as a possible mechanism involved in the observed induction of mitophagy by methionine deficiency.

## Discussion

The precise determination of the role of methionine in farmed fish represents an essential objective for aquaculture industry. In this context, the aim of the present study was to clarify and expand our knowledge on the impact of methionine deficiency on the hepatic oxidative status in rainbow trout and identify the underlying mechanisms.

We found that rainbow trout fed the MD diet did not increase their feed intake (compared with the CTL group) to offset the insufficient amount of methionine provided by this diet. At the end of the 6-week feeding trial, the final body weight as well as the feed efficiency was lower in fish fed the MD diet than in those fed the CTL diet. These results are in accordance with the previously described effects of methionine availability on growth and feed utilisation in fish^24,34–36^. Also, and as expected, fish fed the MD diet exhibited a significant decrease of plasma methionine levels and a concomitant activation of the GCN2-eIF2α pathway in the liver (revealed by the increase of both the phosphorylation of eIF2alpha and the mRNA levels of the two target genes *Asns* and *ddit3)*, indicating that the methionine deficiency was sensed at both tissue and cellular levels, in line to previous findings obtained in rainbow trout^24,37^. In contrast, the phosphorylation of the two mTOR effectors RPS6K1and EIF4EBP1 were not different among the two dietary groups, in agreement with previous results showing that a dietary methionine deficiency of 32% was not able to lower the activation of the mTOR signaling pathway in trout^37^. Overall, these results confirm the previously described effect of methionine availability and provide a relevant material for studying the effect of methionine deficiency on oxidative status in liver.

Here, we observed that dietary methionine deficiency impacted the levels of important markers of the oxidative status (increase of GSH/GSSG ratio associated to a decrease of protein carbonyls) in the liver of trout, without affecting the expression and or/activity of the major antioxidant factors. These results are in line with previous findings demonstrating that methionine restriction decreases mitochondrial ROS production in the liver of rat and pig^21,38^. According to these authors, dietary methionine restriction decreases mitochondrial ROS generation by inhibiting the activity of the respiratory Complex I. In our case, the dietary methionine deficiency induced a more general mitochondrial defect, as shown by the decrease of the level and/or activity of several mitochondrial factors (OXPHOS Complexes I and IV, TIMM23, citrate synthase) in the liver. This is further supported by the increase of the phosphorylation of the energy sensing factor AMPK in the liver of fish fed the MD diet, that indicate a fall in the energy status. However, the relative mitochondrial DNA copy number was not different among the two dietary groups, indicating that the mitochondrial mass was not yet impacted at the monitored time and/or the tested methionine deficiency. Together, these results show that dietary methionine deficiency induced a general mitochondrial defect in the liver of rainbow trout. At first sight, these results might seem surprising in the light of the numerous previous studies having demonstrated the antioxidant properties of methionine in several species including rainbow trout^39–44^. However, we cannot rule out the possibility of an increase of ROS production in methionine deficient condition earlier in the feeding trial that would induce the observed mitochondrial defects and the associated decrease of the oxidative status at 6 weeks.

The accumulation of damaged mitochondria could affect the cell. To maintain cellular homeostasis, eukaryotes have developed a mechanism to eliminate damaged or undesirable mitochondria through macroautophagy of the mitochondria, known as mitophagy^33^. Macroautophagy is an evolutionarily conserved process in eukaryotes by which cytoplasmic cargo sequestered inside double-membrane vesicles or autophagosome are delivered to the lysosome for degradation^45^. Two types of macroautophagy have been identified: non-selective autophagy and selective autophagy which consist in specifically degrading a type of organelle such as mitochondria^46^. Here, we observed by transmission electron microscopy an increase of the number of mitochondria engulfed and/or being engulfed inside autophagosome-related vacuoles in the liver of fish fed the MD diet, supporting a rise of mitophagy in this condition. In order to strengthen these data and precise the underlined mechanisms, we monitored the levels of several factors involved in the PINK1/PARKIN axis which is considered as a major pathway of mitophagy regulation^47^. Briefly, under mitochondrial damage, PINK1 accumulates on mitochondrial outer membrane to further recruit PARKIN and promotes phosphorylation of both S65 on PARKIN’s Ubiquitin-like domain and the conserved S65 residue on ubiquitin itself ^33^. PARKIN mediated ubiquitination of mitochondrial outer membrane proteins would recruit autophagosomal membrane to recognize and surround these marked mitochondria for mitophagosome formation^48^. Several PARKIN substrates have been identified, including Mfn2 involved in mitochondrial fusion^33,49^. After the ubiquitination of Mfn2 by PARKIN, this protein is degraded by the proteasome to arrest mitochondrial fusion^49^. Here, we observed a significant increase of both PARKIN and p-S65-Ub levels, and a concomitant decrease of Mfn2 in the liver of fish fed the MD diet. Overall, the obtained results identified mitophagy as well as the PINK1/PARKIN axis as possible underlying mechanism at play to remove affected mitochondria in the liver of fish fed the methionine deficient diet.

In conclusion, the obtained results showed that feeding rainbow trout with a diet deficient in methionine for 6 weeks results in a drop of growth performances associated to both a general mitochondrial defect and a decrease of the oxidative status in the liver. The obtained results also revealed a sharp increase of mitophagy in these conditions and emphasized the involvement of the PINK1/PARKIN axis in this event. To our knowledge, little if no attention has been paid before to the nutritional regulation of mitophagy and the related mechanisms remain far from being understood and are worth investigating. From a practical aquaculture point of view, we demonstrated that methionine availability in the diet is essential for mitochondrial integrity and can be a trail for understand the decrease of growth in fish fed the MD diet. In the future, another important issue will be to understand how methionine deficiency impact mitochondrial integrity.

## Electronic supplementary material

Supplementary Information
