# Supplementary material for: Dietary methionine deficiency affects oxidative status, mitochondrial integrity and mitophagy in the liver of rainbow trout (Oncorhynchus mykiss)
Source: Sci Rep. 2018 Jul 5;8:10151. doi: 10.1038/s41598-018-28559-8 (PMC6033930; doi:10.1038/s41598-018-28559-8)
Supplement: Supplementary file 1 — Supplementary Information [file 41598_2018_28559_MOESM1_ESM.pdf]

Supplementary information

Dietary methionine deficiency affects oxidative status, mitochondrial integrity and mitophagy in the liver of rainbow trout (Oncorhynchus mykiss)

Sarah Séité, Arnaud Mourier, Nadine Camougrand, Bénédicte Salin, A. Cláudia Figueiredo-Silva, Stéphanie Fontagné-Dicharry, Stéphane Panserat, Iban Seiliez

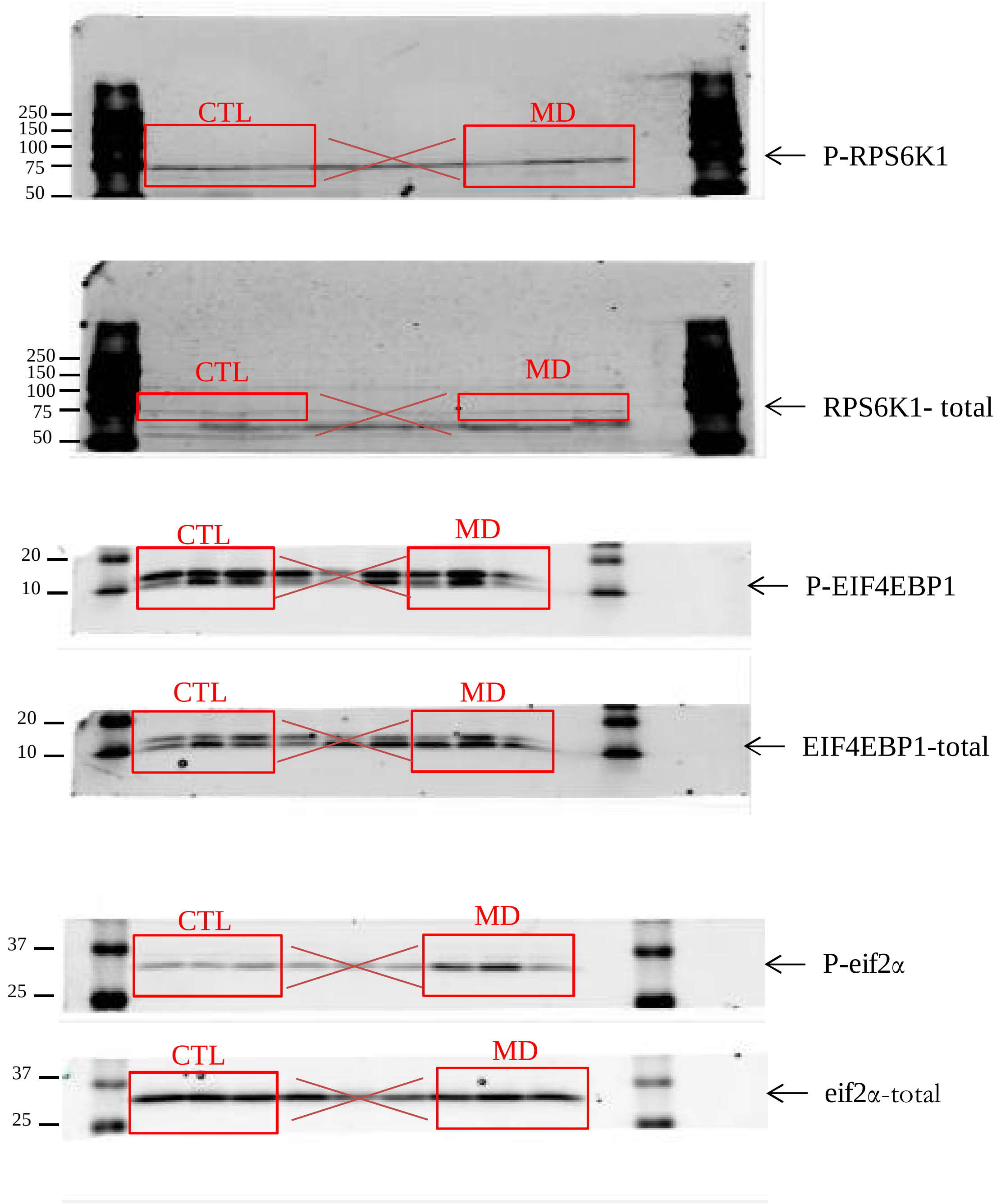

Supplementary Information Figure 1

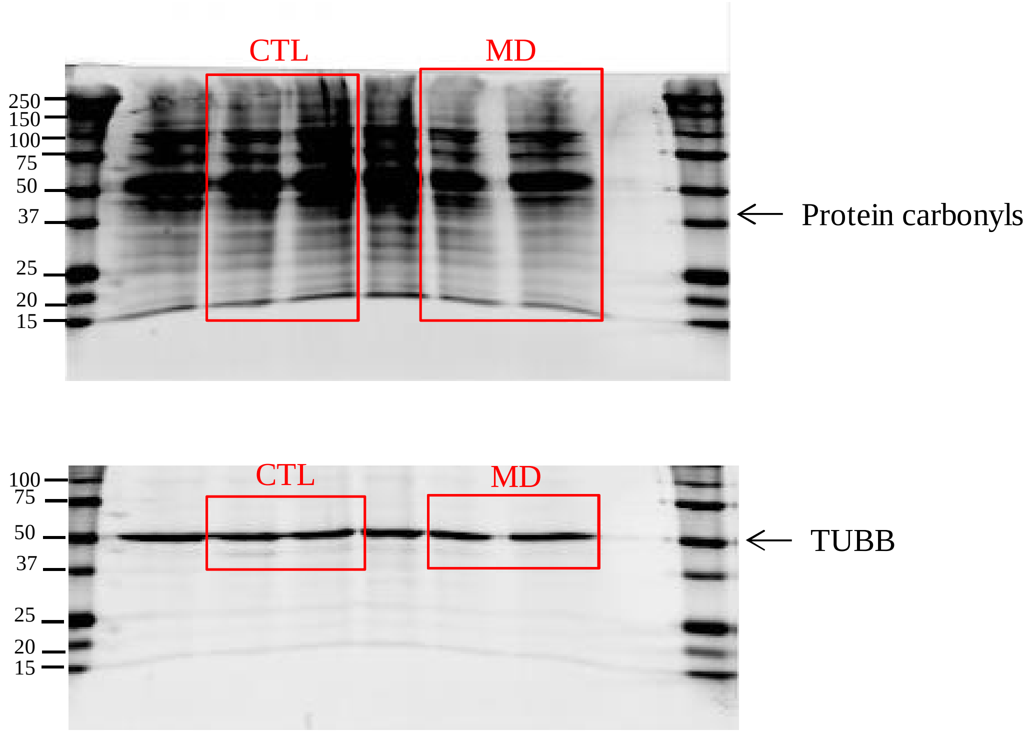

Supplementary Information Figure 2

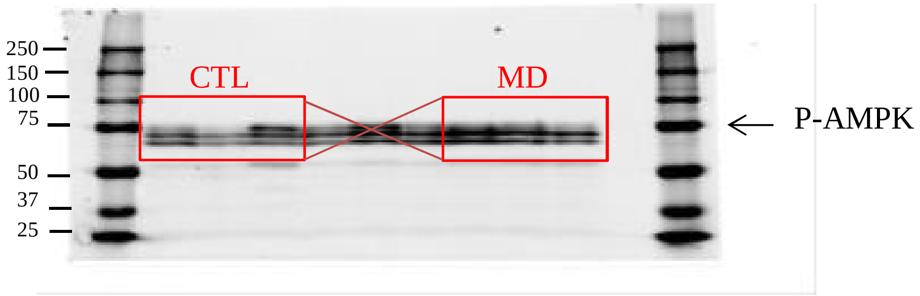

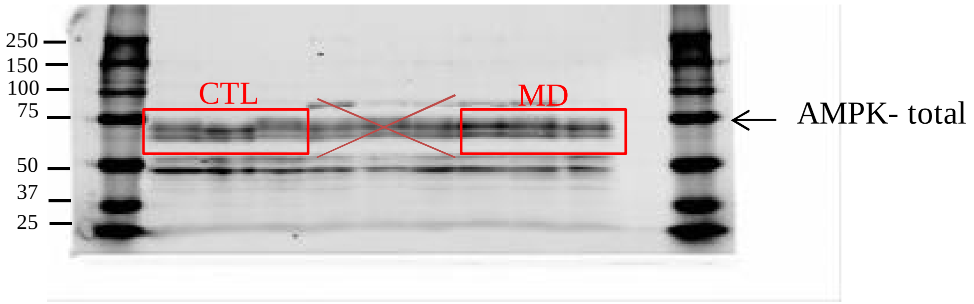

Supplementary Information Figure 4

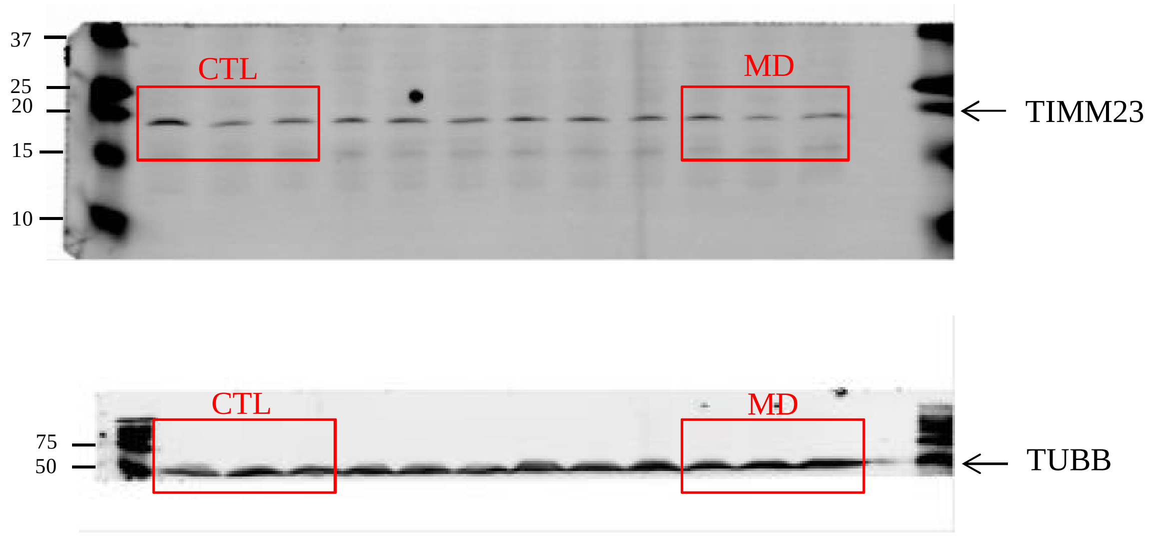

Supplementary Information Figure 5

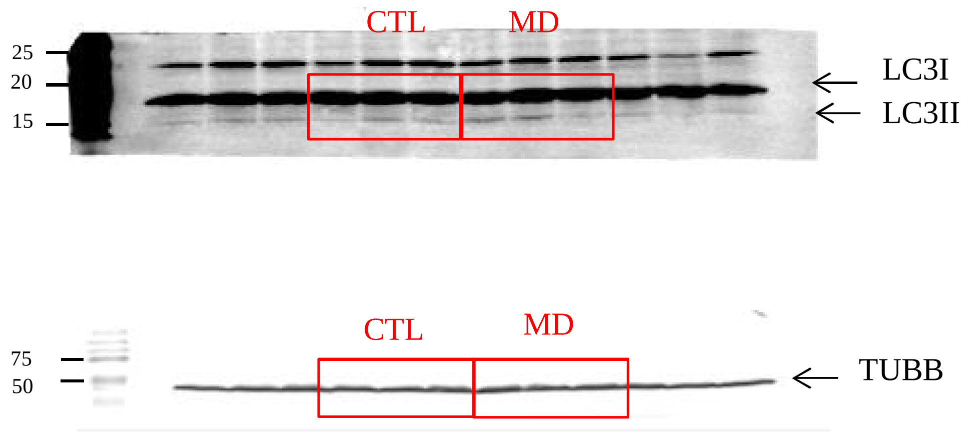

Supplementary Information Figure 6

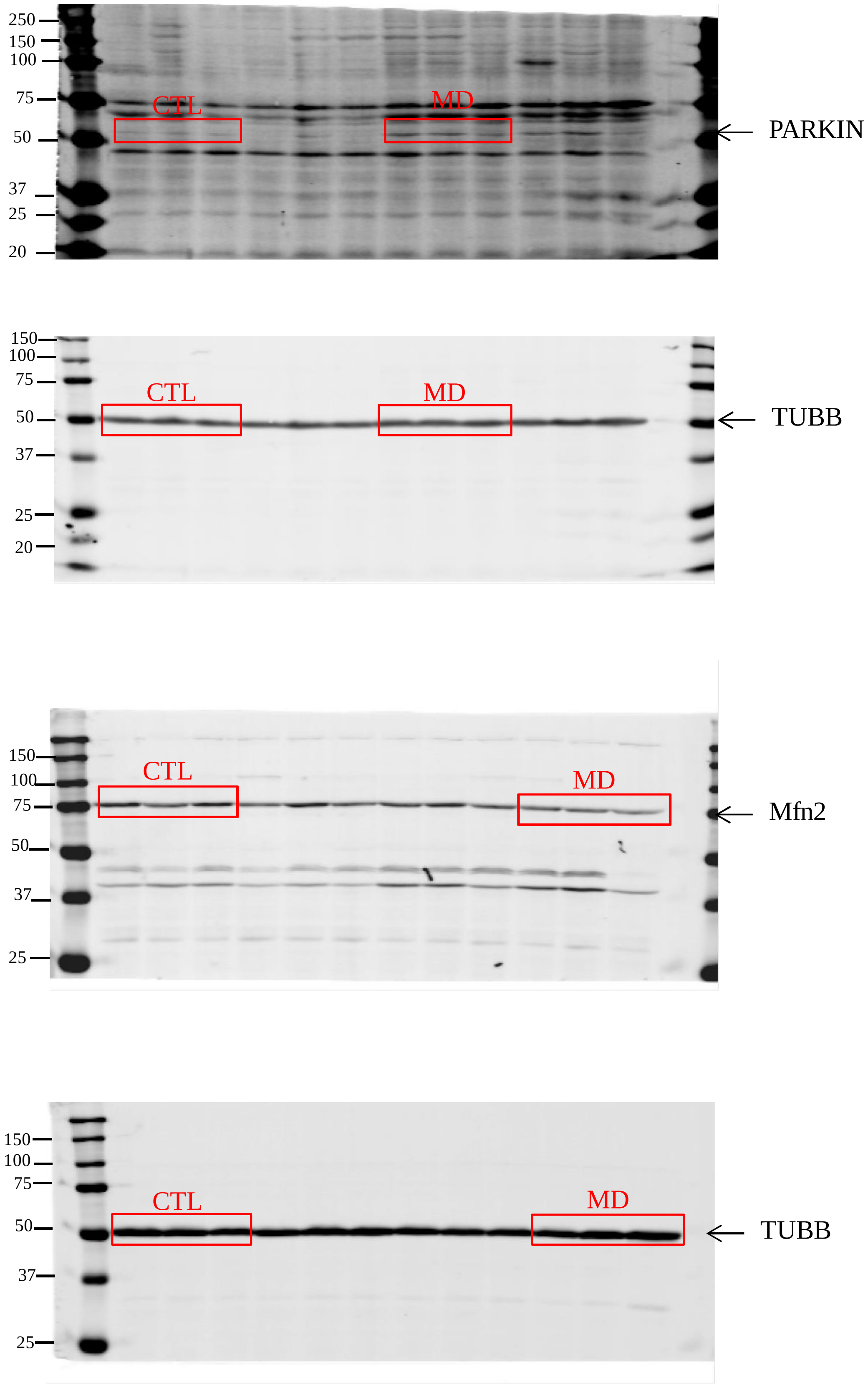

Supplementary Information Figure 7

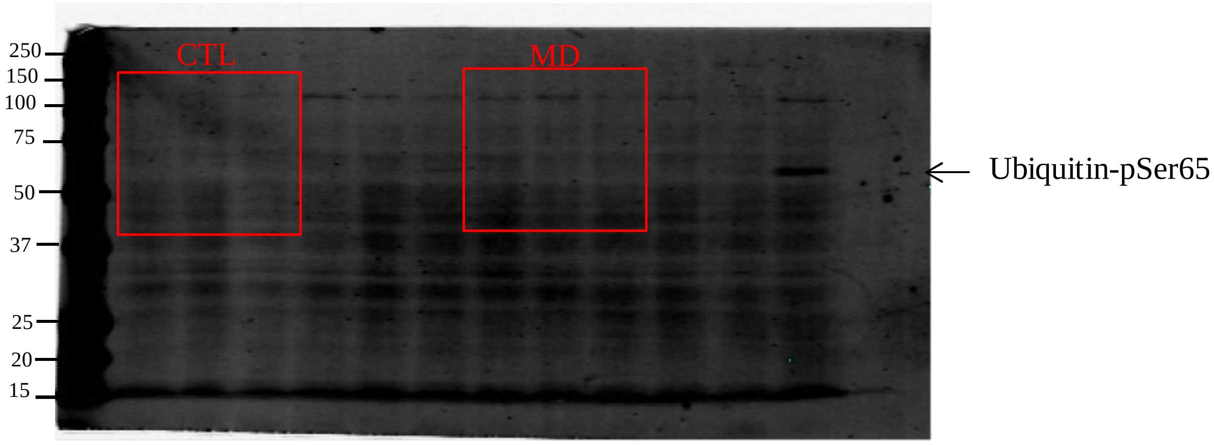

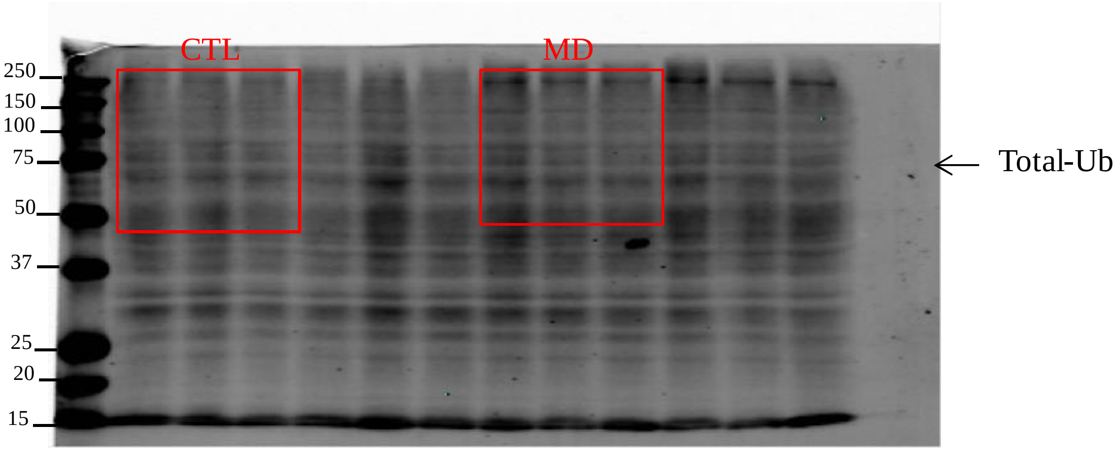

Supplementary Information Figure 7
